# Author Correction: Disentangling the multiorbital contributions of excitons by photoemission exciton tomography

**DOI:** 10.1038/s41467-024-47583-z

**Published:** 2024-04-15

**Authors:** Wiebke Bennecke, Andreas Windischbacher, David Schmitt, Jan Philipp Bange, Ralf Hemm, Christian S. Kern, Gabriele D’Avino, Xavier Blase, Daniel Steil, Sabine Steil, Martin Aeschlimann, Benjamin Stadtmüller, Marcel Reutzel, Peter Puschnig, G. S. Matthijs Jansen, Stefan Mathias

**Affiliations:** 1 I. Physikalisches Institut, Georg-August-Universität Göttingen, Friedrich-Hund-Platz 1, 37077 Göttingen, Germany; 2 Institute of Physics, University of Graz, NAWI Graz, Universitätsplatz 5, 8010 Graz, Austria; 3 Department of Physics and Research Center OPTIMAS, University of Kaiserslautern-Landau, Erwin-Schrödinger-Straße 46, 67663 Kaiserslautern, Germany; 4 Univ. Grenoble Alpes, CNRS, Inst NEEL, F-38042 Grenoble, France; 5 International Center for Advanced Studies of Energy Conversion (ICASEC), University of Göttingen, Göttingen, Germany

**Keywords:** Electronic properties and materials, Surfaces, interfaces and thin films, Electronic structure of atoms and molecules, Excited states

Correction to: *Nature Communications* 10.1038/s41467-024-45973-x, published online 28 February 2024

The original version of the Supplementary Information associated with this Article inadvertently omitted Supplementary Fig. S4. The HTML has been updated to include a corrected version of the Supplementary Information.

### Supplementary information

Updated Supplementary Information

