# Supplementary material for: Disentangling the multiorbital contributions of excitons by photoemission exciton tomography
Source: Nat Commun. 2024 Feb 28;15:1804. doi: 10.1038/s41467-024-45973-x (PMC10899218; doi:10.1038/s41467-024-45973-x)
Supplement: Supplementary file 1 — Supplementary information [file 41467_2024_45973_MOESM1_ESM.pdf]

Disentangling the multiorbital contributions of excitons by photoemission exciton tomography (Supplementary Information)

Wiebke Bennecke,[1] Andreas Windischbacher,[2] David Schmitt,[1] Jan Philipp Bange,[1] Ralf Hemm,[3] Christian S. Kern,[2] Gabriele D'Avino,[4] Xavier Blase,[4] Daniel Steil,[1] Sabine Steil,[1] Martin Aeschlimann,[3] Benjamin Stadtmüller,[3] Marcel Reutzel,[1] Peter Puschnig,[2] G. S. Matthijs Jansen,[1, a)] and Stefan Mathias[1, 5, b)]

[1)] *I. Physikalisches Institut, Georg-August-Universität Göttingen, Friedrich-Hund-Platz 1, 37077 Göttingen, Germany*

[2)] *Institute of Physics, University of Graz, NAWI Graz, Universitätsplatz 5, 8010 Graz, Austria*

[3)] *Department of Physics and Research Center OPTIMAS, University of Kaiserslautern, Erwin-Schrödinger-Straße 46, 67663, Kaiserslautern, Germany*

[4)] *Univ. Grenoble Alpes, CNRS, Inst NEEL, F-38042 Grenoble, France*

[5)] *International Center for Advanced Studies of Energy Conversion (ICASEC), University of Göttingen, Göttingen, Germany*

(Dated: 20 March 2024)

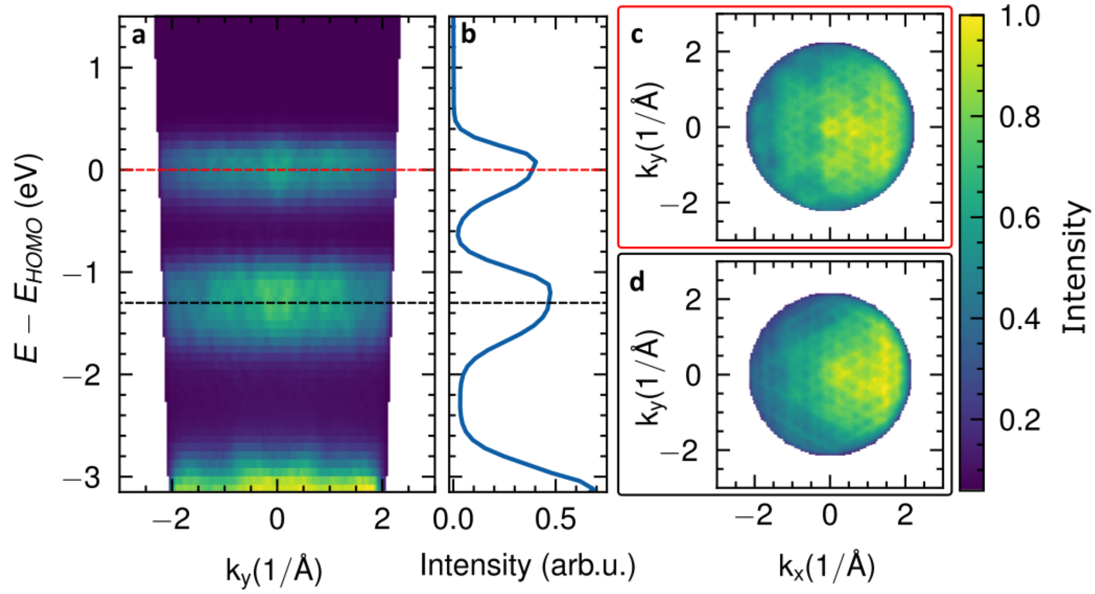

FIG. S1. Static photoelectron momentum microscopy of the multilayer C_{60} sample, taken from the time-resolved data at -1000 fs. The high contrast and line shape of the occupied molecular orbitals as shown in the energy-momentum cut (**a**) and integrated energy distribution curve (**b**) confirm that an influence of the Cu(111) substrate can be ignored. **c, d:** Furthermore, the clear modulation of the HOMO (**c**) and HOMO-1 (**d**) momentum maps confirm the high crystallinity of the multilayer at 80 K[1]

[a)] Electronic mail: gsmjansen@uni-goettingen.de

[b)] Electronic mail: smathias@uni-goettingen.de

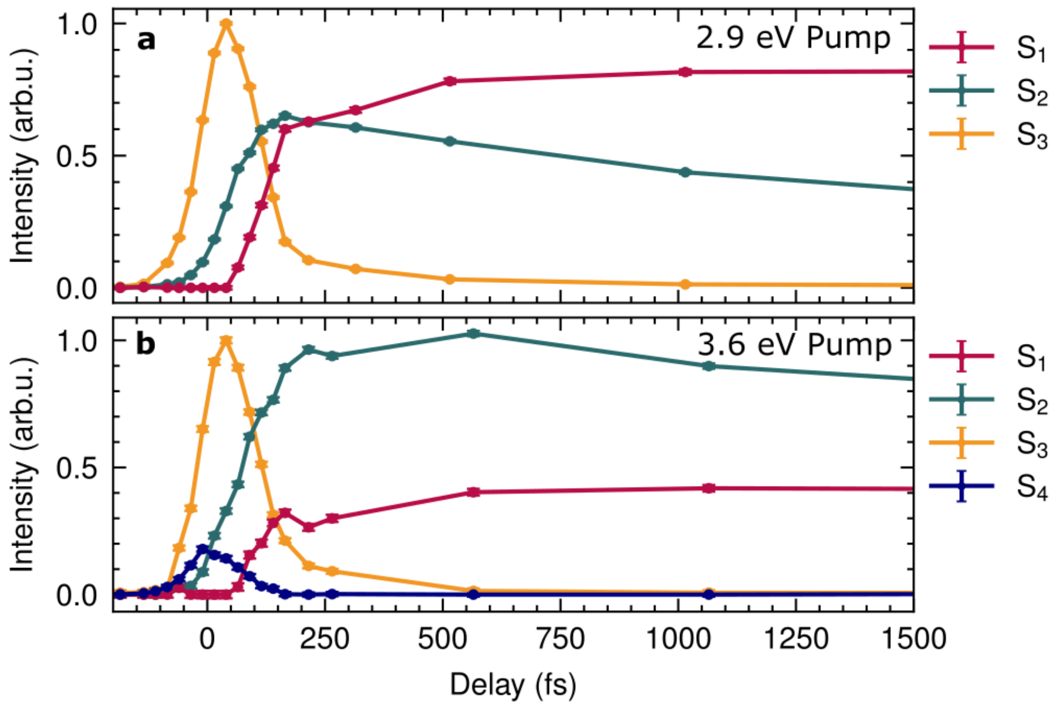

FIG. S2. **a, b:** Exciton-resolved measurement of the femtosecond relaxation dynamics after $h\nu$ =2.9 and $h\nu$ =3.6 eV excitation, respectively. The relative intensities were acquired by fitting the model in Eq. 3 and 4 to the momentum-integrated trPES data. Analysis of the time dependence indicates a sub-50-fs lifetime of the S_4 states. The comparably large S_2 population in the $h\nu = 3.6$ eV measurement can potentially be explained by a relaxation of the S_4 into both the S_3 and S_2 states. Here, the shared Frenkel nature of the S_4 and S_2 excitons might contribute to a relatively fast S_4-S_2 scattering rate, while the charge-transfer nature of the S_3 exciton implies that not only an energetic relaxation but also a spatial charge separation is necessary for the S_4-S_3 process.

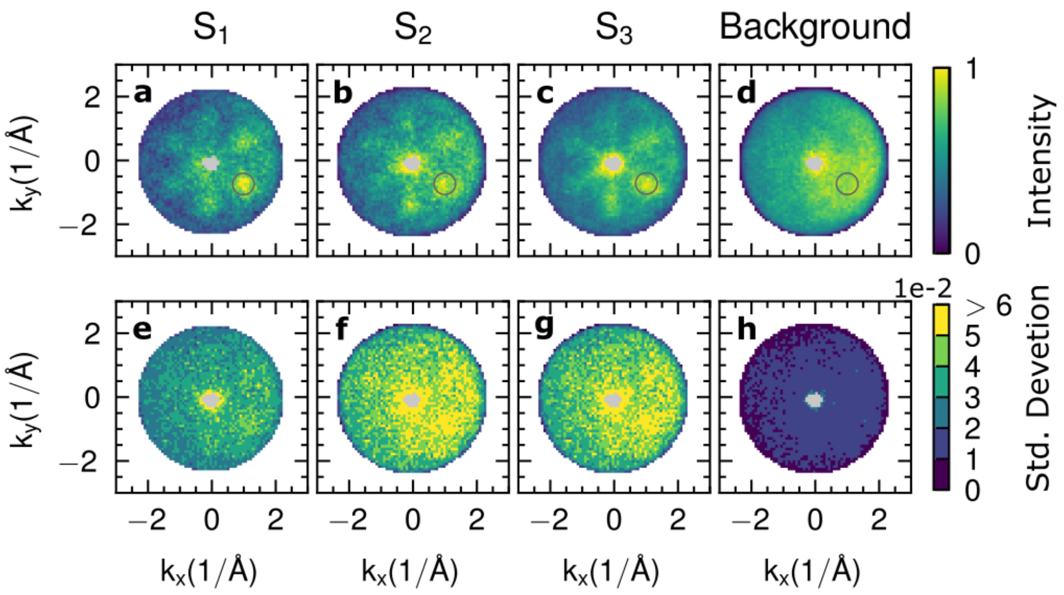

FIG. S3. Amplitude (**a,b,c,d**) and standard deviation (**e,f,g,h**) of the extracted momentum maps of the S_1, S_2, S_3 and the exponential background ($A_{bg}(k)$), respectively. The data are scaled to the mean value inside the circled area.

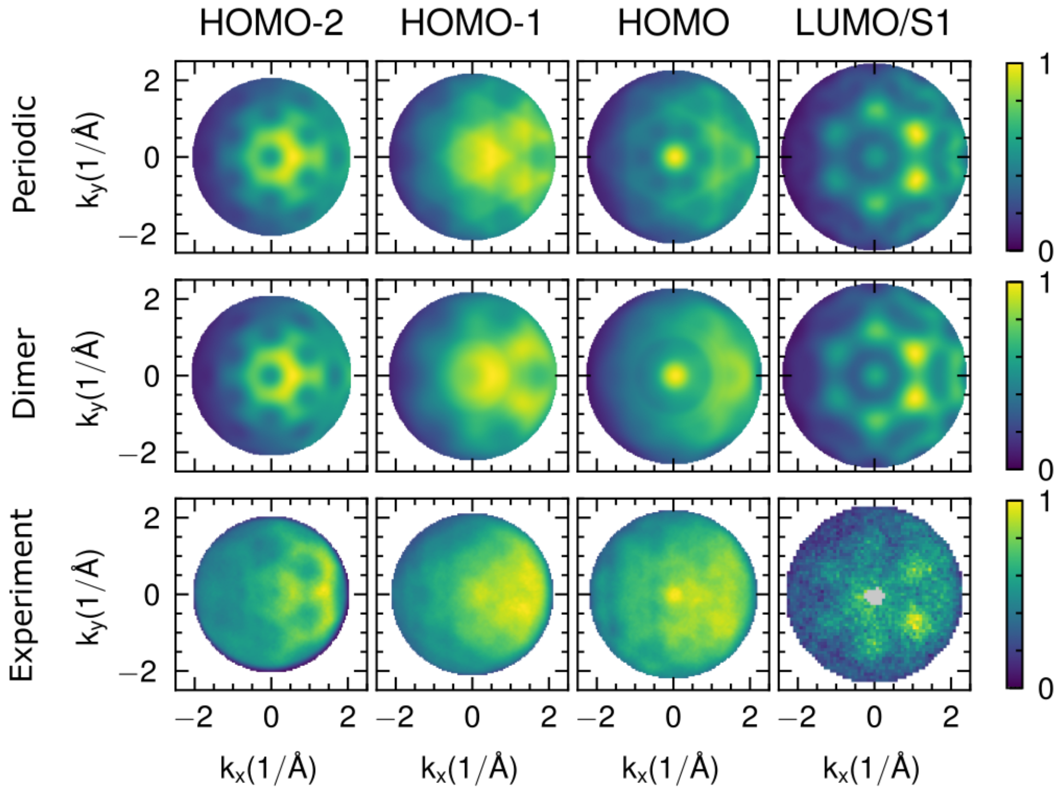

FIG. S4. Comparison of the DFT-calculated momentum maps for a fully periodic monolayer calculation (top row) and for the C_{60} dimer model (middle row) to experiment (bottom row). For the occupied bands, the experimental and theoretical momentum maps were retrieved by integrating over the respective energy ranges, while for the DFT LUMO, we show a comparison to the experimental S_1 momentum fingerprint. The orientation of the 2x2 unit cell was matched to the experimentally observed Brillouin zone (see Fig. 2a and Fig. S1), while the orientation of the molecules was adapted to the observed ARPES momentum fingerprints, i.e., we use static photoemission orbital tomography. The similarity between theory and experiment is most pronounced for the HOMO-2 state, where we observe the strongest contrast in the experiment and no effect of band dispersion. A similar resemblance is seen for the HOMO-1 state when compared with the periodic calculations, while the experimental momentum map of the HOMO does not have clear enough features to be related to the theory. Due to computational complexity, it is not possible at this stage to perform the full BSE calculations on top of the periodic system in an adequate surface (slab) geometry with sufficient amount of vacuum and number of layers. Nevertheless, we can compare the static DFT LUMO and the experimental S_1 momentum map. Same as for the occupied states, the symmetry and general features of the momentum maps are well reproduced. Note that the orientation of the C_{60} molecules in the unit cell was found to be 90° rotated compared to an earlier STM study [Wang et al., 2001][2]. From the qualitative agreement between the experimental and theoretical momentum maps, we conclude that the dimer model provides an accurate description.

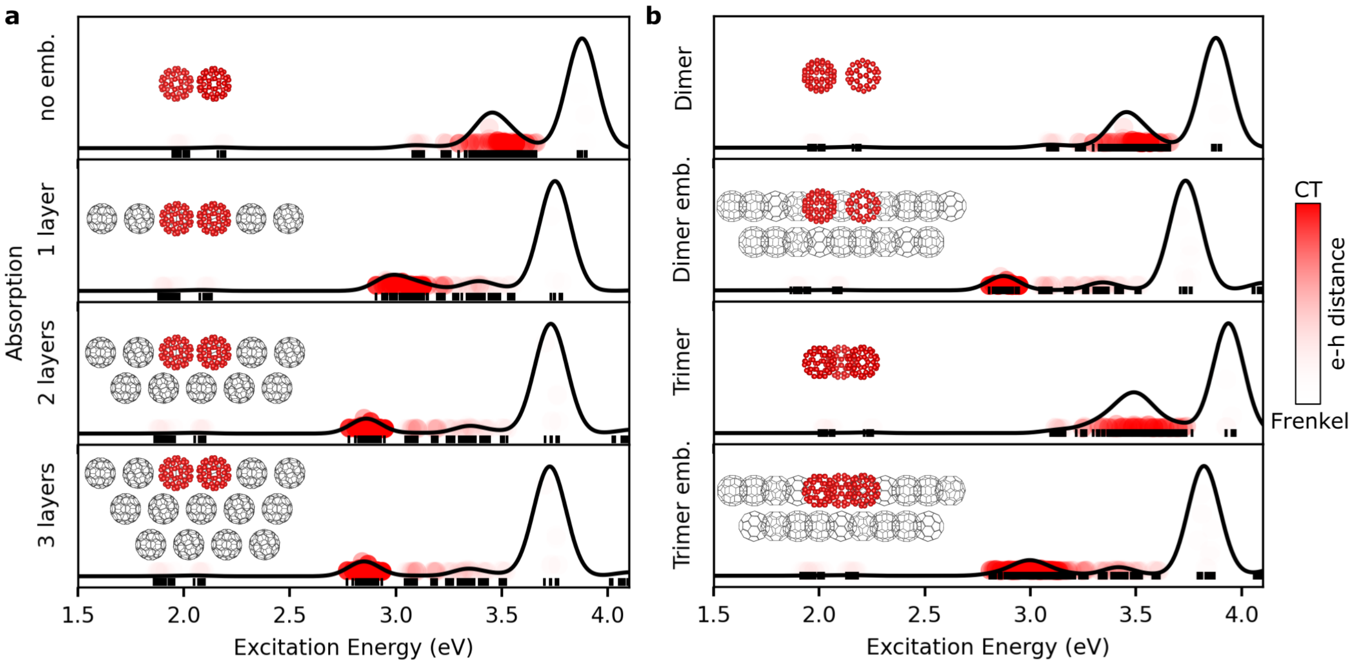

FIG. S5. Convergence test of the embedded GW+BSE calculation with respect to the cluster size and embedding level. a) From the top to bottom panel, full GW+BSE calculations were performed for a C_{60} dimer in increasing embedding conditions. Exciton energies are indicated by the black bars on the horizontal axes. The insets show a cut through the considered 3D multilayer systems. We distinguish charge-transfer (red dots) and Frenkel excitons by their e-h distance. As the embedding changes the dielectric environment and therefore the dielectric screening, charge-transfer excitons are most affected by the procedure. This can be observed, as the band of charge-transfer excitons shifts from 3.4 eV to 2.8 eV when going from 0 to 3-layer embedding. As the exciton spectrum changes only negligibly going from 2 to 3 layers of embedding, we conclude that 2-layer embedding provides an accurate description of the excitons. b) Comparison of the exciton spectra of a C_{60} dimer and a representative C_{60} trimer, at identical levels of embedding. The larger size of the trimer allows for more charge-transfer excitons, but the overall shape of the absorption spectrum does not differ significantly from that of the dimer. As the computational cost of the trimer is significantly larger, higher embedding is currently not feasible. We therefore conclude that, at present, the dimer model leads to the most accurate and reliable results.

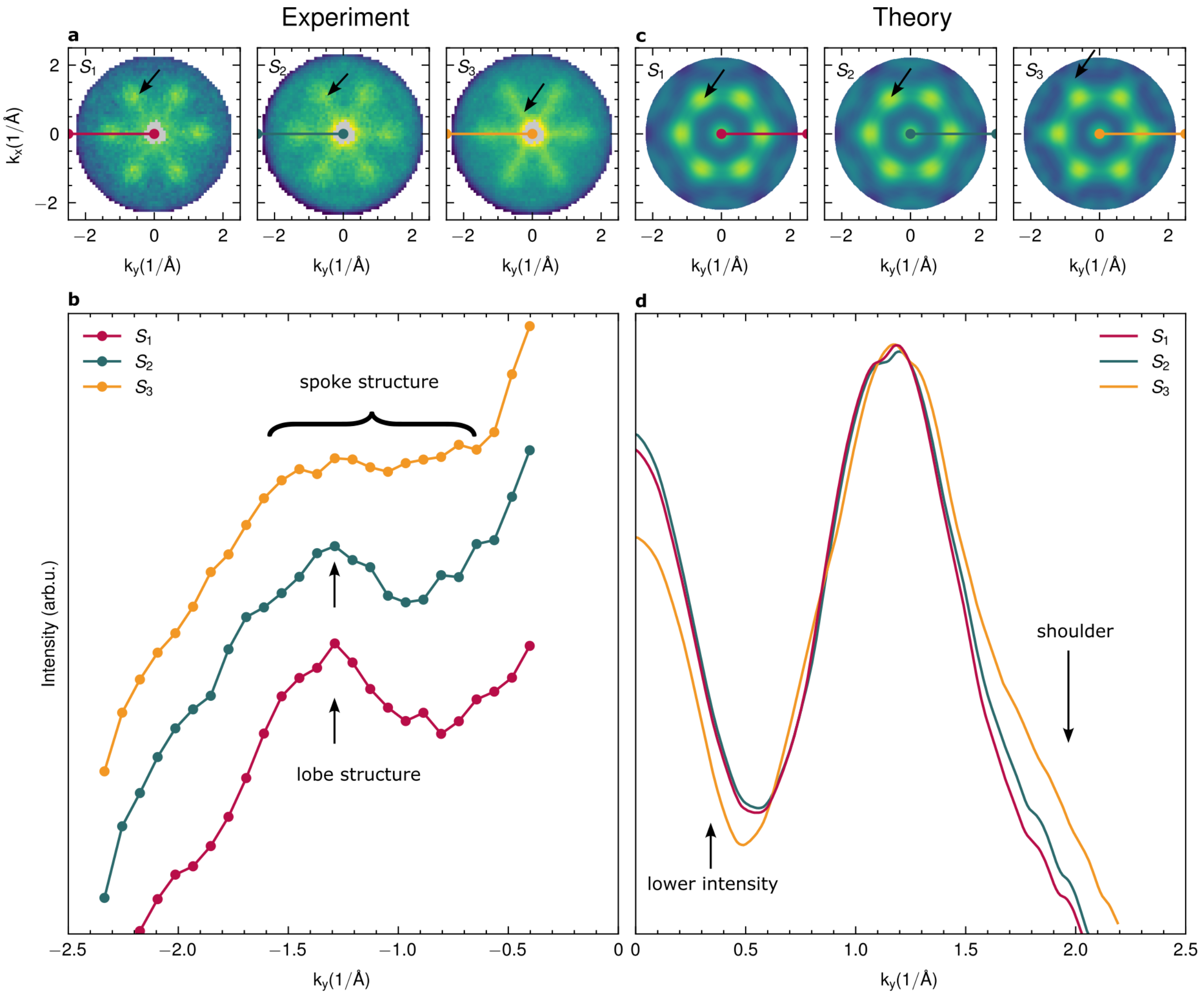

FIG. S6. **a,b** Comparison of the experimentally observed lobe-structure of the $S_1$and S_2 exciton bands in contrast to the spoke-structure of the S_3 exciton band. The main differences are indicated by black arrows in **a**. The photoemission intensity profiles **b** were taken along the lobe and spoke direction as indicated with the colored lines in the symmetrized momentum maps of the S_1, S_2, and S_3 exciton bands in **a** (note that the momentum maps have been rotated by 90° with respect to the main text). The lineouts in the waterfall plot **b** clearly show the lobe structure of S_1 and S_2 as intensity peaks at about ≈ 1.25 Å^{-1}. In contrast, the lineout of the S_3 exciton band does not exhibit a peak, but a nearly uniform intensity distribution between $\approx 0.5 - 1.5$ Å^{-1}, clearly indicating the spoke-structure of S_3. **c,d** A similar analysis was performed for the calculated momentum maps. Here, the S_3 exciton band shows a less pronounced, but still visibly distinct structure in comparison to the S_1 and S_2, as indicated by black arrows in the symmetrized momentum maps **c**. In the lineouts in **d**, the difference is visible by reduced intensity in the $\approx 0.0 - 0.6$ Å^{-1} momentum range and an additional shoulder in the $\approx 1.3 - 2.2$ Å^{-1} momentum range. However, the theoretical model does not reproduce the same spoke-like structure as observed in experiment, as is explained in the main text.

I. REFERENCES

[1] N. Haag, D. Lüftner, F. Haag, J. Seidel, L. L. Kelly, G. Zamborlini, M. Jugovac, V. Feyer, M. Aeschlimann, P. Puschnig, M. Cinchetti, and B. Stadtmüller, "Signatures of an atomic crystal in the band structure of a c_{60} thin film," Phys. Rev. B **101**, 165422 (2020), publisher: American Physical Society.

[2] H. Wang, C. Zeng, B. Wang, J. G. Hou, Q. Li, and J. Yang, "Orientational configurations of the C 60 molecules in the (2 × 2) superlattice on a solid C 60 (111) surface at low temperature," Physical Review B **63**, 085417 (2001).
